# Drowning Complicated by Hypothermia

**DOI:** 10.21980/J8QS7P

**Published:** 2025-01-31

**Authors:** Alexander Close, Jennifer Yee

**Affiliations:** * The Ohio State University, Department of Emergency Medicine, Columbus, OH

## Abstract

**Audience:**

This scenario was developed to educate emergency medicine residents on the diagnosis and management of two concurrent conditions: drowning and hypothermia.

**Introduction:**

Patients who present after drowning may have delayed respiratory compromise without immediate radiographic pathological findings, highlighting the need for continued observation. The presentation and management of patients with hypothermia depends on multiple factors, including core temperature. Emergency physicians should be aware of hypothermia’s underlying pathophysiology, associated dysrhythmias, and different warming methods.

**Educational Objectives:**

At the conclusion of the simulation session, learners will be able to:

1. Obtain a relevant focused history, including circumstances of drowning and/or cold exposure.
2. Outline different clinical presentations of hypothermia, loosely correlated with core temperature readings.
3. Discuss management of hypothermia, including passive external rewarming, active external rewarming, active internal rewarming, and extracorporeal blood rewarming.
4. Discuss pathophysiology of drowning.
5. Identify appropriate disposition of patients who present after drowning.
6. Identify appropriate disposition of hypothermic patients.

**Educational Methods:**

This session was conducted using high-fidelity simulation, followed by a debriefing session and discussion about the diagnosis, differential, and management of both drowning and hypothermia. Debriefing methods may be left to the discretion of participants, but the authors have utilized advocacy-inquiry techniques. In this technique, the facilitators describe something they observed in the case, outline their reasoning as a facilitator why this observation was important or why they had questions, and then ask the learners to share their frame of reference at the time. An example: “I heard someone say that both chest tubes should be placed on the left, but then another resident said ‘I disagree.’ No one paused to come to a consensus. I’m wondering why this wasn’t explored further in real time. Tell me more.” This scenario may also be run as a structured interview case.

**Research Methods:**

Our residents were provided a survey at the completion of the debriefing session so they might rate different aspects of the simulation, as well as provide qualitative feedback on the scenario. The local institution’s simulation center’s electronic feedback form is based on the Center of Medical Simulation’s Debriefing Assessment for Simulation in Healthcare (DASH) Student Version Short Form^1^ with the inclusion of required qualitative feedback if an element was scored less than a 6 or 7.

**Results:**

Seventeen learners filled out a feedback form. This session received a majority of 6 and 7 scores (consistently effective/very good, and extremely effective/outstanding, respectively) other than four 5 scores.

**Discussion:**

This is a cost-effective method for reviewing hypothermia and drowning. The case may be modified for appropriate audiences, such as simplifying the case to either drowning or hypothermia. The setting of the emergency department may also be changed to reflect different available resources (academic center or freestanding emergency department), such as the absence or presence of ZOLL catheters or ability to activate an extracorporeal membrane oxygenation (ECMO) team.

**Topics:**

Medical simulation, drowning, hypothermia, environmental emergencies, emergency medicine.

## USER GUIDE

[Table t1-jetem-10-1-s43]

**Table t1-jetem-10-1-s43:** 

List of Resources:	
Abstract	43
User Guide	45
Instructor Materials	48
Operator Materials	62
Debriefing and Evaluation Pearls	64
Simulation Assessment	70

**Learner Audience:**

Interns, Junior Residents, Senior Residents

**Time Required for Implementation:**

**Instructor Preparation:** 30 minutes

**Time for case:** 20 minutes

**Time for debriefing:** 40 minutes

**Recommended Number of Learners per Instructor:** 3–4

**Topics:**

Medical simulation, drowning, hypothermia, environmental emergencies, emergency medicine.

**Objectives:**

By the end of this simulation session, the learner will be able to:

1. Obtain a relevant focused history, including circumstances of drowning and/or cold exposure.
2. Outline different clinical presentations of hypothermia, loosely correlated with core temperature readings.
3. Discuss management of hypothermia, including passive external rewarming, active external rewarming, active internal rewarming, and extracorporeal blood rewarming.
4. Identify appropriate disposition of hypothermic patients.
5. Discuss pathophysiology of drowning.
6. Identify appropriate disposition of patients who present after drowning.

### Linked objectives and methods

Moderately and severely hypothermic patients who have had a drowning event require aggressive evaluation and management. This includes obtaining a relevant focused history, including circumstances of drowning and/or cold exposure (Objective 1). Participants should be able to outline different clinical presentations of hypothermia, loosely correlated with core temperature readings (Objective 2) and be able to describe methods of passive external, active external, active internal, and extracorporeal blood rewarming (Objective 3). Hypothermic patients should be appropriately dispositioned to the medical intensive care unit with consideration for the need for ECMO (Objective 4). To address the drowning aspect of the case, participants should be able to discuss pathophysiology of drowning (Objective 5) and identify appropriate disposition of patients who present after drowning (Objective 6). This high-fidelity simulation scenario allows learners to reinforce drowning and hypothermia management skills in a psychologically-safe learning environment, and then receive formative feedback on their performance.

### Recommended pre-reading for instructor

We recommend that instructors review literature regarding hypothermia and drowning, including presenting signs/symptoms, diagnosis, and management. Suggested readings include materials listed under the “References/suggestions for further reading” section below.

### Results and tips for successful implementation

This simulation was written for emergency medicine residents to be performed as a high-fidelity simulation scenario. It also may be used as a structured interview or as a case for learners interested in wilderness medicine. We ran this case with six small groups of emergency medicine residents.

Our residents rated the debriefing using Harvard’s Debriefing Assessment for Simulation in Healthcare assessment ( https://harvardmedsim.org/debriefing-assessment-for-simulation-in-healthcare-dash/). This session received a majority of 6 and 7 scores (consistently effective/very good, and extremely effective/outstanding, respectively) other than four 5 scores. Examples of these 5 scores were as follows: to the assessment statement, “The instructor helped me see how to improve or how to sustain good performance,” the learner commented, “I think we got in the weeds a bit on the discussion about decision making in a case that ultimately had a lot of room for reasonable differences in practice.” For the statement, “The instructor identified what I did well or poorly-and why,” the resident wrote, “Group feedback is very helpful in these simulations but I think providing more individual feedback would be helpful as well.” The lowest average score was 6.375 for, “The instructor identified what I did well or poorly - and why.” The highest average score was 6.76 for, “During the simulation, the instructor maintained an engaging context for learning.” The form also includes an area at the end for general feedback about the case. An illustrative example of feedback includes, “[I] really enjoyed this case - particularly helpful to combine drowning and hypothermia.”

A chest x-ray confirming placement of right-sided tube thoracostomy may be presented that reflects the multiple options of laterality and placement of chest tubes, such as: “Your chest x-ray shows tube placement as such, but on the opposite side,” for left-sided chest tubes. Alternatively, if multiple chest tubes are placed, the facilitator may state, “Your chest x-ray shows that all chest tubes are placed in a manner similar to this representative image,” allowing the residents to still evaluate for appropriate placement without immediate radiographic findings of iatrogenic injury.

In the interest of time to allow the case to progress and to preserve any overlying “skin” of the task trainer for subsequent groups, the authors suggest having the residents place one to two chest tubes in a task trainer (if available) rather than double bilateral chest tubes. Facilitators may also choose to just observe one chest tube placement and allow the residents to subsequently verbalize the number and location of additional chest tubes.

One recurring theme that we noticed was learners gave the patient a much lower Glascow Coma Scale (GCS) than expected and felt he was not able to protect his airway. Due to his ongoing hypoxia and altered mentation, they elected to prioritize intubation early. In several instances, all efforts to rewarm the patient were halted in order to intubate the patient, causing delays in rewarming. But causing the patient to cough more vigorously, to verbalize responses, and to open his eyes to stimulation may reassure learners to explore rewarming options first.

## Supplementary Information



## Figures and Tables

**Table t2-jetem-10-1-s43:** 

#1	Complete blood count (CBC)
#2	Basic metabolic panel (BMP)
#3	Hepatic function panel
#4	Partial Thromboplastin Time (PTT)
#5	Prothrombin Time (PT)/International Normalized Ratio (INR)
#6	Troponin
#7	Lactate
#8	Venous blood gas
#9	Urinalysis
#10	Electrocardiogram
#11	Chest X-ray
#12	Chest X-ray (intubated)
#13	Chest X-ray (intubated with right-sided chest tube) #14 Computed Tomography (CT) head
#15	CT cervical spine

**Table t3-jetem-10-1-s43:** 

**Vitals:**	Heart Rate (HR) 54	Blood Pressure (BP) 96/68
	Respiratory Rate (RR) 12	Temperature (T) 83.0°F
	Oxygen Saturation (O_2_Sat) 90% on a non-rebreather mask at 15 liters/minute	

**Table t4-jetem-10-1-s43:** 

*Complete blood count (CBC)*	
White blood count (WBC)	9.1 × 1000/mm^3^
Hemoglobin (Hgb)	15.1 g/dL
Hematocrit (HCT)	43%
Platelet (Plt)	300 × 1000/mm^3^

**Table t5-jetem-10-1-s43:** 

*Basic metabolic panel (BMP)*	
Sodium	138 mEq/L
Potassium	4.0 mEq/L
Chloride	99 mEq/L
Bicarbonate (HCO_3_)	26 mEq/L
Blood Urea Nitrogen (BUN)	9 mg/dL
Creatinine (Cr)	0.6 mg/dL
Glucose	105 mg/dL
Calcium	8.8 mg/dL

**Table t6-jetem-10-1-s43:** 

*Hepatic function panel*	
Total bilirubin	0.8 mg/dL
Direct bilirubin	0.2 mg/dL
Albumin	4.1 g/dL
Alkaline Phosphate	58 units/L
Aspartate Aminotransferase (AST)	23 units/L
Alanine Aminotransferase (ALT)	22 units/L

**Table t7-jetem-10-1-s43:** 

*Coagulation Profile*	
Partial Thromboplastin Time (PTT)	30.0 seconds
Prothrombin Time (PT)	13.0 seconds
International Normalized Ratio (INR)	1.0

**Table t8-jetem-10-1-s43:** 

*Troponin*	10 ug/mL

**Table t9-jetem-10-1-s43:** 

*Lactic acid*	1.7 mmol/L

**Table t10-jetem-10-1-s43:** 

*Arterial blood gas (ABG), pre-intubation*	
pH	7.31
paCO2	50 mmHg
paO2	32 mmHg
HCO3	25 mmol/L
SaO2	90% on FiO2 50%

**Table t11-jetem-10-1-s43:** 

*Urinalysis*	
Color	clear yellow
Spec gravity	1.015
Protein	negative
Glucose	negative
Ketones	negative
Bilirubin	negative
Hemoglobin	negative
Leukocyte esterase	negative
Nitrite	negative
Red blood cells (RBC)	0–5/high powered field (HPF)
White blood cells (WBC)	0–5/HPF
Squamous epithelial	0–5/HPF

**Figure f1-jetem-10-1-s43:**
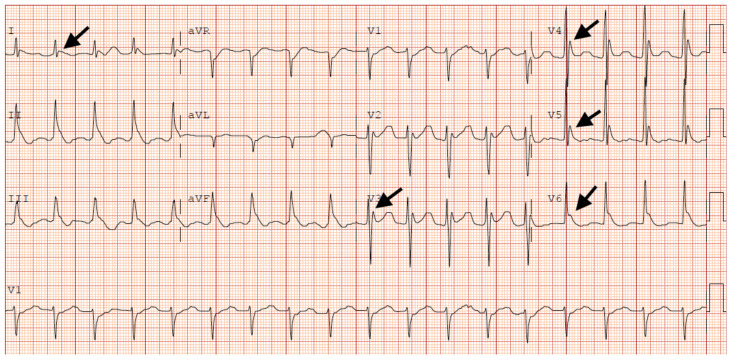


**Figure f2-jetem-10-1-s43:**
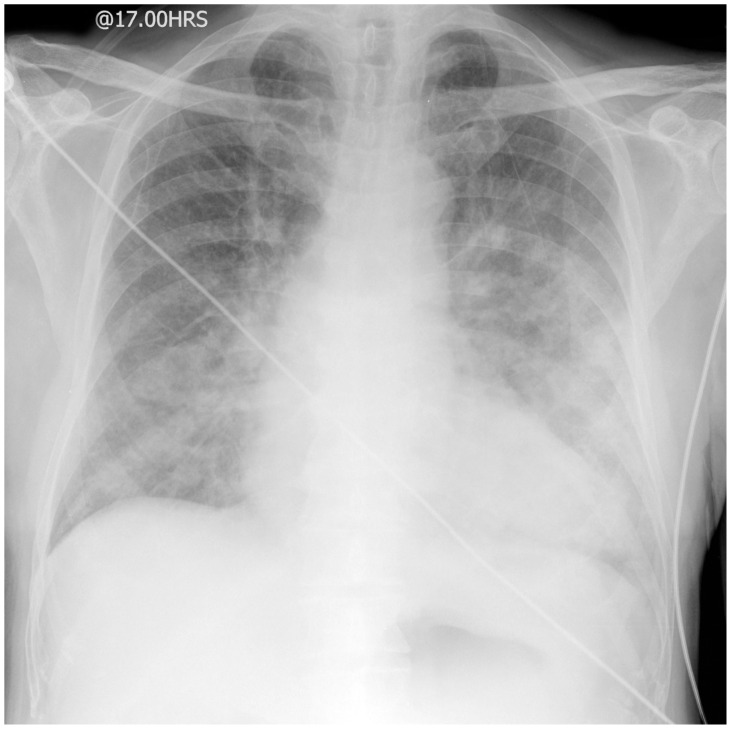


**Figure f3-jetem-10-1-s43:**
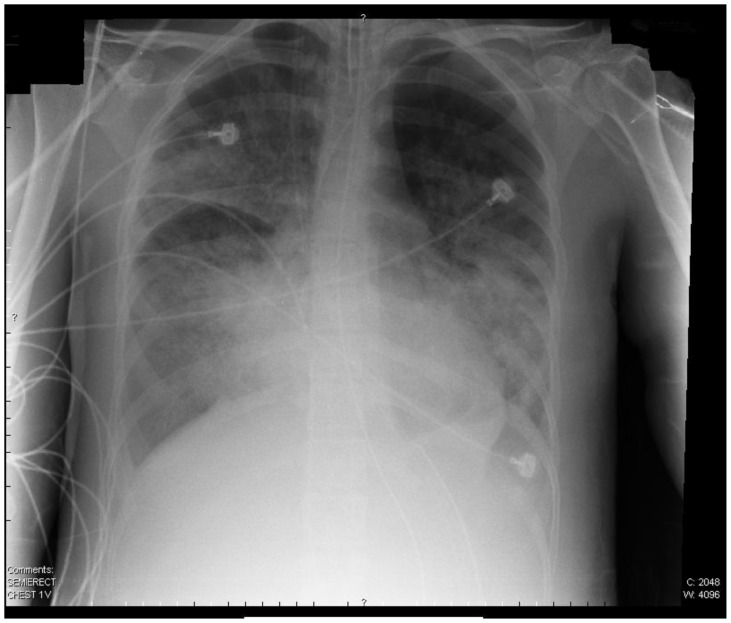


**Figure f4-jetem-10-1-s43:**
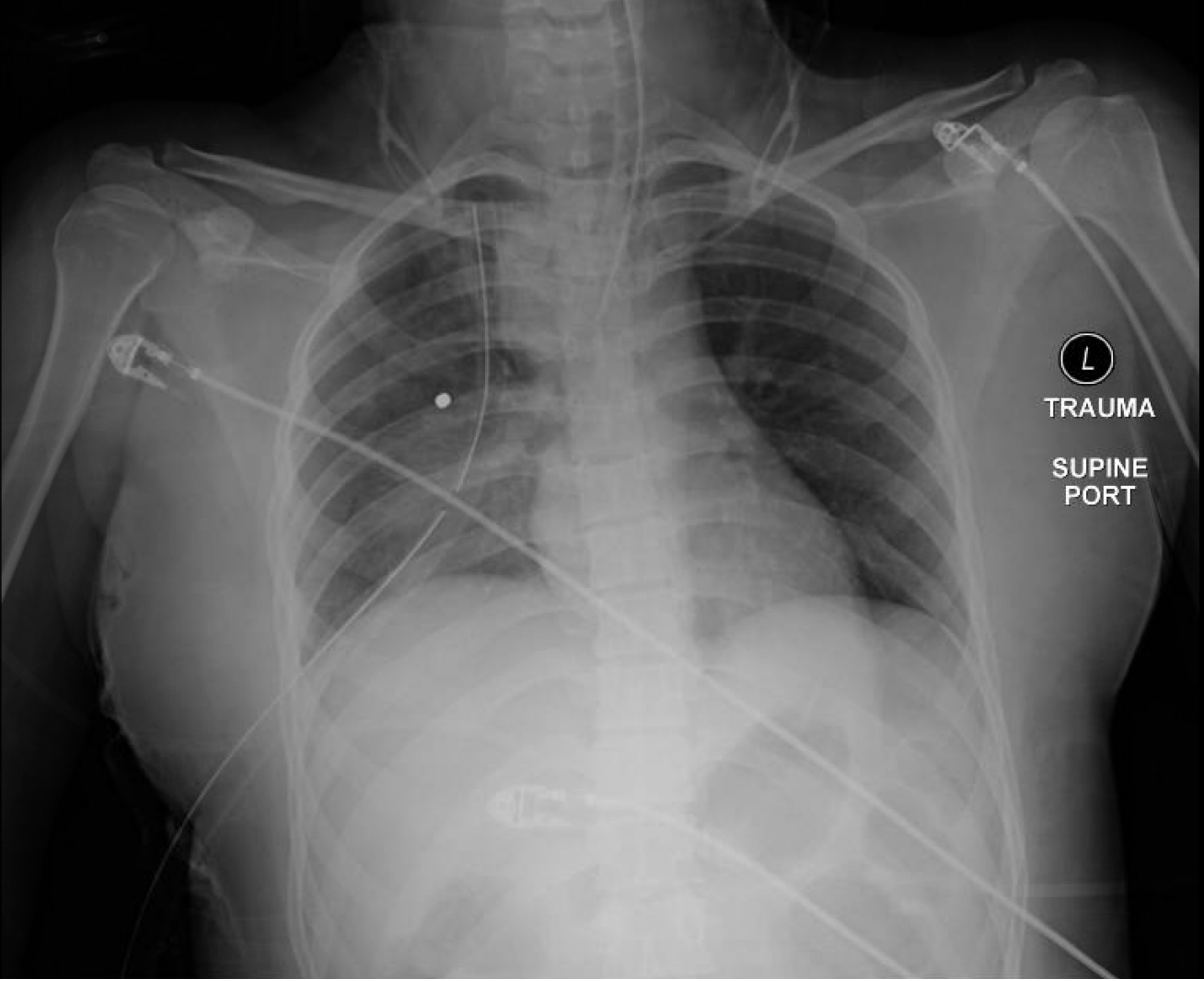


**Figure f5-jetem-10-1-s43:**
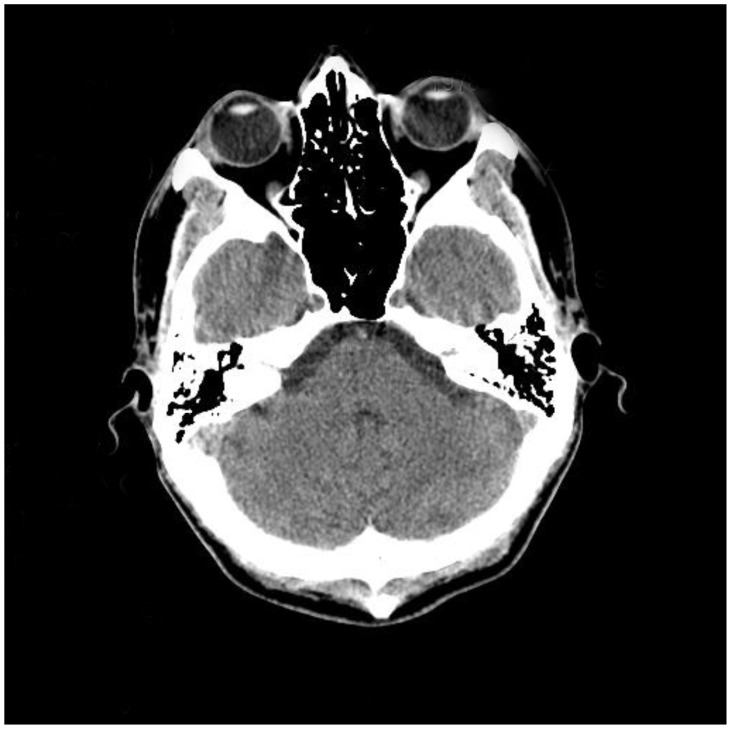


**Figure f6-jetem-10-1-s43:**
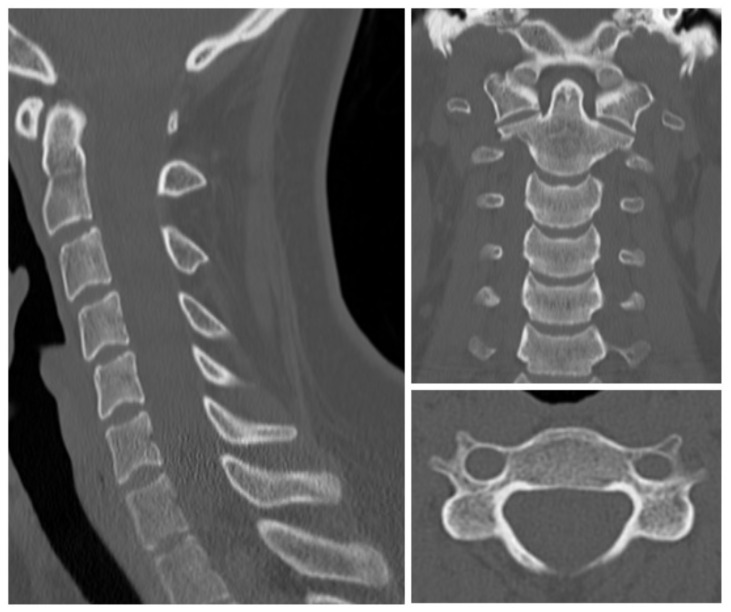


**Table t12-jetem-10-1-s43:** SIMULATION EVENTS TABLE:

Minute (state)	Participant action/ trigger	Patient status (simulator response) & operator prompts	Monitor display (vital signs)
0:00 (Baseline)	Team enters patient’s room in the emergency department.	Participants should begin by placing the patient on a monitor, obtaining history from EMS/partner, and performing an undressed physical exam. Temperature will not be demonstrated until team asks for rectal temperature or temperature-sensing foley catheter.	A: T 83°F HR 54 BP 96/68 RR 12 O_2_ sat 90% on non-rebreather mask at 15 liters/minute
3:00 (State 2)	Team should recognize the patient is hypothermic and hypoxic. Bair Hugger, warm fluids, and warmed humidified air should be initiated. Patient should be intubated. Labs, EKG, and CXR should be ordered.	If the team does not place ipsilateral chest tubes and irrigate warm fluids through them AND intubate the patient by minute 9, go to state #3, Unstable V-Tach. If the team DOES place ipsilateral chest tubes and irrigate warm fluids through them by minute 9 but DOES NOT intubate, go to state #4, Warmed but More Altered. If the team hyperventilates the patient (RR>10), go to state #3, Unstable V-Tach. If the team places ipsilateral chest tubes and irrigates warm fluids through them and ventilates patient appropriately, (RR 6–10) go to state #5, Intubated and Warmed.	T 80.5°F HR 44 BP 86/42 RR 8 O_2_ sat 87% on non-rebreather mask at 15 liters/minute (or 90% on BiPAP CPAP) OR O_2_ sat 95% if intubated on 100% Fio2
9:00 (State 3)	The monitor displays ventricular tachycardia.	The team should begin ACLS management and identify hypothermia and hypoxia as a reversible cause of cardiac arrest. They should defibrillate once and withhold further defibrillation attempts until patient reaches 86°F. After 2 rounds of ACLS, learners should recognize return of spontaneous circulation. Go to state #5, Intubated and Warmed.	T 78.8°F HR 0 BP 0/0 RR 0 EtCO2 10
9:00 (State 4)	The patient is warmed, but is now unresponsive (GCS 5). Glasgow Coma Scale 5 (Eyes: 2 Verbal: 2 Motor: 1)	If the team does not begin to intubate by minute 10, go to state #3, Unstable V-Tach. If the team begins to intubate by minute 10, (RR 6–10) go to state #5, Intubated and Warmed once intubated.	T 83°F HR 54 BP 96/68 RR 6 O_2_ sat 70% on non-rebreather mask at 15 liters/minute (or 80% on BiPAP/CPAP)
13:00 (State 5)	Participants should contact ICU for admission.	End Case	T 83°F HR 54 BP 96/68 RR 12 O_2_ sat 95% Intubated on 100% Fio2

**Table t13-jetem-10-1-s43:** 

Stages of Hypothermia: Modified Staging System	Temperature in Celsius	Temperature in Fahrenheit	Consciousness
1: Mild	32–35	90–95	Conscious
2: Moderate	28–32	82–90	Impaired consciousness
3: Severe	<28	<82	Unconscious
4: Hypothermic cardiac arrest (can occur at temperatures > 32C/90F but most likely to happen in Severehypothermia)	<32	<90	Vital signs absent

**Table:** 

Stage 1	Remove wet clothes, if sheltered	Passive external
	Heat room, if sheltered	Passive external
	Blankets	Passive external
	Warm drinks	Active internal
Stage 2	**Start Core Temperature Monitoring**	
	Heating blankets (trunk initially)	Active external
	Bair Hugger (trunk initially)	Active external
	Warm humidified air via heated high flow, facemask, or endotracheal tube	Active internal
	Heated fluids (Not very effective)	Active internal
Stage 3	ZOLL catheter insertion	Active internal
	Gastric lavage (Not very effective)	Active Internal
	Peritoneal lavage	Active internal
	Thoracic (used only when in arrest or ZOLL is unavailable or not effective)	Active internal
	ECMO or dialysis (used if patient is not rewarming with other measures or in arrest)	Active internal

**Figure:**
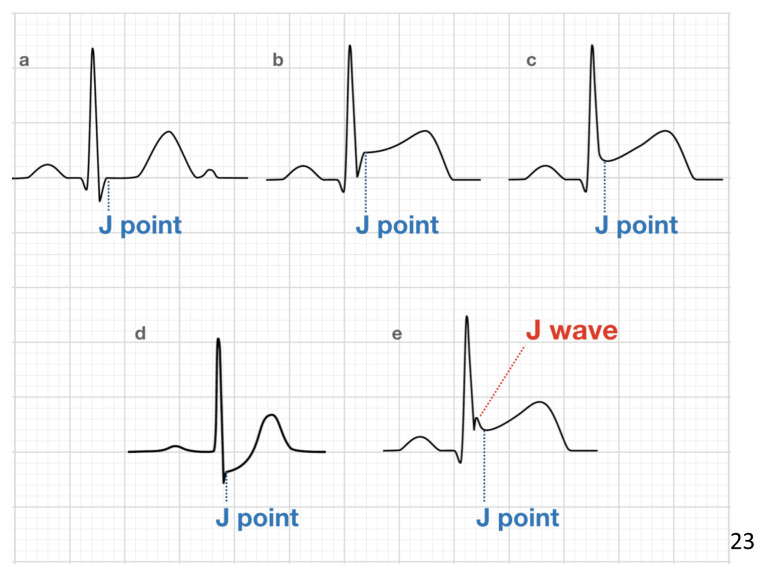


**Table t15-jetem-10-1-s43:** 

**Critical Actions:** Place patient on monitor and obtain a full set of vitals, including temperature Obtain point-of-care glucose Place two large bore IV catheters Initiate heated high-flow oxygen delivery Remove all wet clothing Place a Bair-hugger Perform a physical exam specifically evaluating for potential traumatic injuries Place a temperature-sensing foley catheter Place two right-sided chest tubes for thoracic irrigation Update partner on diagnosis and planned disposition Admit to the Intensive Care Unit (ICU) with ECMO capabilities	0:00

**Table t16-jetem-10-1-s43:** 

	Milestone	Did not achieve level 1	Level 1	Level 2	Level 3
1	**Emergency Stabilization (PC1)**	□ Did not achieve Level 1	□ Recognizes abnormal vital signs	□ Recognizes an unstable patient, requiring intervention Performs primary assessment Discerns data to formulate a diagnostic impression/plan	□ Manages and prioritizes critical actions in a critically ill patient Reassesses after implementing a stabilizing intervention
2	**Performance of focused history and physical (PC2)**	□ Did not achieve Level 1	□ Performs a reliable, comprehensive history and physical exam	□ Performs and communicates a focused history and physical exam based on chief complaint and urgent issues	□ Prioritizes essential components of history and physical exam given dynamic circumstances
3	**Diagnostic studies (PC3)**	□ Did not achieve Level 1	□ Determines the necessity of diagnostic studies	□ Orders appropriate diagnostic studies. Performs appropriate bedside diagnostic studies/procedures	□ Prioritizes essential testing Interprets results of diagnostic studies Reviews risks, benefits, contraindications, and alternatives to a diagnostic study or procedure
4	**Diagnosis (PC4)**	□ Did not achieve Level 1	□ Considers a list of potential diagnoses	□ Considers an appropriate list of potential diagnosis May or may not make correct diagnosis	□ Makes the appropriate diagnosis Considers other potential diagnoses, avoiding premature closure
5	**Pharmacotherapy (PC5)**	□ Did not achieve Level 1	□ Asks patient for drug allergies	□ Selects an medication for therapeutic intervention, consider potential adverse effects	□ Selects the most appropriate medication and understands mechanism of action, effect, and potential side effects Considers and recognizes drug-drug interactions
6	**Observation and reassessment (PC6)**	□ Did not achieve Level 1	□ Reevaluates patient at least one time during case	□ Reevaluates patient after most therapeutic interventions	□ Consistently evaluates the effectiveness of therapies at appropriate intervals
7	**Disposition (PC7)**	□ Did not achieve Level 1	□ Appropriately selects whether to admit or discharge the patient	□ Appropriately selects whether to admit or discharge Involves the expertise of some of the appropriate specialists	□ Educates the patient appropriately about their disposition Assigns patient to an appropriate level of care (ICU/Tele/Floor) Involves expertise of all appropriate specialists
9	**General Approach to Procedures (PC9)**	□ Did not achieve Level 1	□ Identifies pertinent anatomy and physiology for a procedure Uses appropriate Universal Precautions	□ Obtains informed consent Knows indications, contraindications, anatomic landmarks, equipment, anesthetic and procedural technique, and potential complications for common ED procedures	□ Determines a back-up strategy if initial attempts are unsuccessful Correctly interprets results of diagnostic procedure
20	**Professional Values (PROF1)**	□ Did not achieve Level 1	□ Demonstrates caring, honest behavior	□ Exhibits compassion, respect, sensitivity and responsiveness	□ Develops alternative care plans when patients’ personal beliefs and decisions preclude standard care
22	**Patient centered communication (ICS1)**	□ Did not achieve level 1	□ Establishes rapport and demonstrates empathy to patient (and family) Listens effectively	□ Elicits patient’s reason for seeking health care	□ Manages patient expectations in a manner that minimizes potential for stress, conflict, and misunderstanding. Effectively communicates with vulnerable populations, (at risk patients and families)
23	**Team management (ICS2)**	□ Did not achieve level 1	□ Recognizes other members of the patient care team during case (nurse, techs)	□ Communicates pertinent information to other healthcare colleagues	□ Communicates a clear, succinct, and appropriate handoff with specialists and other colleagues Communicates effectively with ancillary staff

## INSTRUCTOR MATERIALS

**Case Title:** Drowning Complicated by Hypothermia

**Case Description & Diagnosis (short synopsis):** The patient is a 19-year-old male who presents by Emergency Medical Services (EMS) to a community hospital emergency department after falling through the ice while playing ice hockey on a pond. He was initially unresponsive but shortly after began moaning and spontaneously coughing. EMS placed the patient on a non-rebreather for hypoxia.

Once in the emergency department, he is noted to be altered with a Glasgow Coma Scale of 10 with sinus bradycardia and hypoxia. He can intermittently answer yes or no questions. The team should put the patient on the cardiac monitor, remove the patient’s wet cold clothes and perform a trauma evaluation. A rectal temperature or a temperature-sensing foley will confirm hypothermia. Once hypothermia is confirmed, active external and internal methods should be employed, including delivery of heated high flow oxygen. ZOLL catheters are not available and the community hospital does not have ECMO capabilities, so the team should perform right-sided thoracic irrigation with two ipsilateral chest tubes.

Patient will have increased heart rate/blood pressure/temperature with active internal rewarming. His oxygenation status will remain unchanged on a non-rebreather, eventually becoming unresponsive and hypoxic until intubation is required. If the patient does not undergo active internal rewarming, he will decompensate to ventricular tachycardia until active internal rewarming is initiated. The patient’s partner should be updated on the overall clinical course and eventual transfer to a medical intensive care unit with ECMO capabilities.

**Equipment or Props Needed:**

- High fidelity simulation manikin
- Velcro (“tear away”) clothes for the manikin, if available
- Ice packs to place on the manikin’s clothes between each iteration of the scenario
- Bedside ultrasound machine, if available
- Angiocatheters for large bore peripheral intravenous access = 18 gauge, 20 gauge
- Tube thoracostomy task trainer
- Two chest tubes
- Tube thoracostomy placement kit
- Cardiac monitor
- Pulse oximetry
- IV pole
- Bilevel Positive Airway Pressure (BiPAP) mask
- Non-rebreather mask
- Bair hugger
- High-flow oxygen cannula, if available
- Warm blankets
- Foley catheter
- Normal saline (1 liter ×2)
- Lactated Ringer’s (1 liter ×2)
- Simulated medications with labeling: etomidate, succinylcholine, rocuronium, fentanyl, midazolam

**Actors needed:**

Primary nurse, patient’s partner. Faculty may call in overhead as the admitting physician.

**Stimulus Inventory:** [Table t2-jetem-10-1-s43]

**Background and brief information:** The confederate nurse says to the team upon arrival to the simulation bay: “Patient is a 19-year-old male who presented by EMS after falling through the ice while playing ice hockey on a pond.”

**Initial presentation:** 19-year-old male in wet cold street clothes, lying supine on the gurney with a non-rebreather mask in place. Further history may be obtained by EMS providers and/or partner.

- Past medical history: none
- Past surgical history: none
- Medications: none
- Allergies: none
- Social history: no tobacco use, alcohol use, or illicit drug use
- Family history: noncontributory
- Emergency department vital signs:
  ○ Heart Rate (HR) – 54 beats per minute
  ○ Respiratory rate – 12 respirations per minute
  ○ Temperature – 83° Fahrenheit (F) – only displayed once residents ask for a rectal temperature or a temperature-sensing foley catheter
  ○ Blood Pressure – 96/68 millimeters of mercury (mmHg)
  ○ Pulse oximetry – 90% on a non-rebreather mask at 15 liters/minute
- Weight: 75 kilograms (kg)

Assessment: Lying supine, coughing, non-rebreather mask in place

**How the scene unfolds:** Patient is brought via EMS to a community hospital emergency department after falling through the ice. Participants should elicit more information from EMS and/or the patient’s partner, including that the patient fell through the ice on a pond at some point when they were left alone for five minutes. The partner dragged him out of the water, noting that he was initially unresponsive before spontaneously coughing and moaning. EMS placed the patient on a non-rebreather mask for hypoxia to 80% on room air.

The team should put the patient on the cardiac monitor, remove the patient’s wet cold clothes, and perform a trauma evaluation. When an axillary temperature is not reading, they should request a rectal temperature or a temperature-sensing foley. Once hypothermia is confirmed, active external and internal methods should be employed, including delivery of heated high flow oxygen. ZOLL catheters are not available and the community hospital does not have ECMO capabilities, so the team should perform right-sided thoracic irrigation with two ipsilateral chest tubes. The patient’s mental status, oxygenation, and ability to protect his airway should be repeatedly reassessed.

Patient will have increased heart rate/blood pressure/temperature with active internal rewarming. His oxygenation status will remain unchanged on a non-rebreather, eventually becoming unresponsive and hypoxic until intubation is required. If the patient does not undergo active internal rewarming, he will decompensate to ventricular tachycardia until active internal rewarming is initiated. The patient’s partner should be updated on clinical course and eventual transfer disposition to a medical intensive care unit with ECMO capabilities.

**Critical actions:**

1. Place patient on monitor and obtain a full set of vitals, including temperature
2. Obtain point-of-care glucose
3. Place two large bore IV catheters
4. Initiate heated high-flow oxygen delivery
5. Remove all wet clothing
6. Place a Bair-hugger
7. Perform a physical exam specifically evaluating for potential traumatic injuries
8. Place a temperature-sensing foley catheter
9. Place two right-sided chest tubes for thoracic irrigation
10. Update partner on diagnosis and planned disposition
11. Admit to the Intensive Care Unit (ICU) with ECMO capabilities

**Case Title:** Drowning Complicated by Hypothermia

**Chief Complaint:** Fell through the ice[Table t3-jetem-10-1-s43]

**General Appearance:** Lying supine in the gurney in cold, wet street clothes, non-rebreather mask in place

**Primary Survey:**

- **Airway:** coughing, can answer simple yes/no questions intermittently
- **Breathing:** bradypnea, rales present bilaterally
- **Circulation:** bradycardic, 1+ symmetric radial and dorsalis pedis pulses bilaterally, capillary refill 4–5 seconds

**History:**

- **History of present illness:** History provided by partner and/or EMS. Patient was found underwater in a home pond by partner. Patient had been playing ice hockey on a pond while his partner watched from the house. The partner stepped away from the window for five minutes; when he returned to the window, he saw a hole in the ice with the patient nowhere to be found. He found him under the water and subsequently dragged him out. Patient was initially unresponsive for several minutes, but had aroused with coughing upon EMS arrival. If asked, the pond is at least 10 feet deep, and it is currently about 20°F outside. His glucose was 100 mg/dL for EMS.
- **Past Medical History**: none
- **Past Surgical History:** none
- **Patient’s Medications:** none
- **Allergies:** none
- **Social History:** no tobacco, alcohol or illicit drug use
- **Family History:** noncontributory

**Vital Signs:**

- HR – 54
- Resp rate – 12
- Temp – 83.0° F (only displayed once rectal temperature or temperature-sensing foley catheter requested)
- BP – 96/68
- Pulse ox – 90% on a non-rebreather, 15 liters/min
- Weight: 75 kg

**Assessment:** Lying supine in the gurney in cold, wet street clothes, non-rebreather mask in place

**Secondary Survey/Physical Examination:**

- **General Appearance**: Lying supine in the gurney in cold wet street clothes, non-rebreather mask in place, appears stated age. Intermittent coughing.
- **HEENT**
  ○ Head: wet, otherwise within normal limits
  ○ Eyes: dilated sluggish pupils, otherwise within normal limits
  ○ Ears: within normal limits
  ○ Nose: within normal limits
  ○ Throat / oropharynx: within normal limits
- **Neck**: within normal limits.
- **Heart**: regular rhythm, bradycardic. Otherwise within normal limits
- **Lungs**: rales bilaterally and symmetric. Bradypnea.
- **Abdominal/GI**: within normal limits
- **Genitourinary**: within normal limits
- **Rectal**: within normal limits
- **Extremities**: within normal limits
- **Neuro**: Glasgow Coma Scale 9 (Eyes: 2 Verbal: 3 Motor: 4)
- **Skin**: damp and cold, capillary refill reduced to 4–5 seconds.
- **Lymph**: within normal limits
- **Psych**: within normal limits

[Table t4-jetem-10-1-s43] [Table t5-jetem-10-1-s43] [Table t6-jetem-10-1-s43] [Table t7-jetem-10-1-s43] [Table t8-jetem-10-1-s43] [Table t9-jetem-10-1-s43] [Table t10-jetem-10-1-s43] [Table t11-jetem-10-1-s43]

*Electrocardiogram*

Van Eukelom, Jon. Osborn Waves in a Severely Hypothermic Patient. In: JetEM. https://jetem.org/osborn_waves_hypothermicpt/. Open access[Fig f1-jetem-10-1-s43]

*Chest X-ray*

Frank Gaillard, Jeremy Jones. AP portable CXR of a patient in acute pulmonary oedema. In: Wikimedia Commons. File:AP portable CXR of a patient in acute pulmonary oedema.jpg - Wikimedia Commons. Public Domain.[Fig f2-jetem-10-1-s43]

*Chest X-ray (Intubated)*

Samir. Chest X-ray of a patient with ARDS. In: Wikimedia Commons. File:ARDS X-Ray.jpg - Wikimedia Commons. Public domain.[Fig f3-jetem-10-1-s43]

*Chest X-ray (intubated with right-sided chest tube)*

Hemothorax Pre and Post Chest Tube CXR. In: JetEM. Hemothorax Pre and Post Chest Tube CXR - JETem. Public domain.[Fig f4-jetem-10-1-s43]

*CT head*

Ciscel, Andrew. Head CT scan. In: Wikimedia Commons. https://commons.wikimedia.org/wiki/File:Head_CT_scan.jpg. Public domain.[Fig f5-jetem-10-1-s43]

*CT cervical spine*

Mikael Häggström. Computed tomographs of normal cervical vertebrae (thumbnail). In: Wikimedia Commons. https://commons.wikimedia.org/wiki/File:Computed_tomographs_of_normal_cervical_vertebrae_(thumbnail).jpg. Public domain.[Fig f6-jetem-10-1-s43]

## OPERATOR MATERIALS

[Table t12-jetem-10-1-s43]

**Diagnosis:**

Drowning complicated by hypothermia

**Disposition:**

Transfer to a medical ICU with ECMO capabilities

## DEBRIEFING AND EVALUATION PEARLS

### Drowning Complicated by Hypothermia

**Drowning**

Definition by the World Health Organization^1^: “the process of experiencing respiratory impairment from submersion/immersion in a liquid”

- Subclassified into “drowning with mortality” and “drowning without mortality”
  ○ Do not use the terms “near-drowning,” “wet” or “dry” drowning
- Greater than 370,000 deaths caused by drowning worldwide and around 4,000 deaths in the United States annually 2,3
- Incidence for drowning is highest in males, African Americans, patients of low socioeconomic status, and children one to five years old 4,5
  ○ Young children typically drown while bathing or after falling into swimming pools

Pathophysiology

Drowning with mortality 6,7

1. Typically starts with panic, breath holding, followed by air hunger
2. Followed by a reflex inspiration, likely resulting in aspiration
3. Irritation to the lower respiratory tract causes coughing, likely worsening aspiration
4. Results in hypoxia, causing a loss in consciousness, and cardiac arrest
  Water dilutes surfactant and increases the permeability of alveolar membrane 8,9
  - Can lead to non-cardiogenic pulmonary edema and acute respiratory distress syndrome (ARDS)
  - Occurs in both salt and fresh water
  - ARDS can develop despite benign initial imaging, requires close monitoring

Initial Management of Drowning

1. Perform an airway assessment
  Intubation indications are the same as patients who have not had a submersion
2. Oxygen supplementation
3. Get vitals
  Evaluate for concomitant hypothermia
4. Cervical immobilization and CT head are not necessary in all cases because they are rare without concerning history or associated diving^10^

Management and Disposition of Drowning Without Mortality Based on GCS and Oxygenation

If GCS greater than or equal to 13 and greater than or equal to 95% oxygenation saturation^11^,^12^

- Observe for six hours
  Pulmonary symptoms can develop anytime within this time frame
- No need for laboratory studies and radiographs
- If there are no rales, rhonchi, wheezing, retractions, and if the oxygen saturation is greater than 95%, the patient can be discharged with good return precautions

If GCS Less than 13 11–13

- Admit patient
- Supplemental oxygen and support
- Intubate if the patient cannot maintain more than 60 mmhg oxygen or (80 mmhg in children) on an arterial blood gas (ABG) on BiPAP, or if the patient cannot protect their airway
- Get a chest X-ray, ABG and CBC, BMP, EKG to evaluate for pulmonary aspiration event No need for prophylactic antibiotics for aspiration event 14
- Admit to ICU for continuous cardiac monitoring and frequent vital reassessments
- Get a repeat X-ray after 4 hours

Scene Management of Drowning

1. Ensure scene safety for yourself/rescuers
2. Remove the person from the water
3. Ask someone to call 9-1-1
4. Check for a pulse (pulse may be weak/difficult to detect)
5. Begin chest compressions with rescue breathing (morbidity often due to hypoxemia)
6. Remove wet clothes
7. Place defibrillator pads

**Hypothermia**

Body temperature <35° Celsius (95° F)

- Around 1500 deaths are caused by hypothermia in the United States annually 15
- It is caused by increased heat loss or decreased production of heat
- Risk factors include being subject to cooler temperatures, vasodilation from drugs, carbon monoxide, alcohol intoxication
- Secondary hypothermia; metabolic or internal cause of hypothermia
  Causes: hypopituitarism, hypothyroidism, hypoadrenalism, hypoglycemia, burns, rashes that are erythematous--eczema, ichthyosis, psoriasis, or sepsis or trauma, bleeding
  Treatment: antibiotics, thiamine, hydrocortisone, levothyroxine, hyperglycemia
- After drop; a drop in blood pressure and temperature after initial rewarming due to reduced peripheral vasoconstriction and increased blood flow to colder peripheral extremities

[Table t13-jetem-10-1-s43]

16

Shivering is an unreliable gauge of patient temperature. It is present and absent across a wide range of temperature.^16^–^17^

18–20

Mechanisms of Heat Transfer

Conduction: Direct transition of heat from substance to substance

Convection: Heat transfer from a surface to a fluid as it flows over that surface

Radiation: All objects lose heat via radiation based on intrinsic properties

Evaporation: As fluids transition to gas, heat is lost from underlying structures

Initial Management of Hypothermia

1. Check and make sure the thermometer can actually get a reading
2. Check blood glucose
3. Rewarm the patient appropriately
4. Get an EKG
5. Consider BMP, chest x-ray, lipase, PT, INR
  - Coagulopathy that occurs during hypothermia is not reflected in laboratory testing because coagulation studies are performed at 37°C.
  - Blood gas studies that are rewarmed are not reflective of patient’s true acid base status
6. Vasoactive drugs should not be administered until temperature is 30°C or higher.^19^

Respiratory Status and Support in Hypothermia

- Acid base status is not accurately reflected if the sample is rewarmed
- Titrate respiratory support based on a noncorrected blood gas sample with the goal of pH of 7.4 and PaCO2 of 40 21,22
- Hypothermic patients have a component alkalosis secondary to Le Châtelier's principle. (As water cools, more H+ and HCO3 convert to water at equilibrium). This occurs both intracellularly and extracellularly. Bypass surgery studies show an association between alkalosis and reduced fibrillation threshold in hypothermic patients. This is thought to be related to maintaining the transmembrane pH potential. Blood gas analyses are performed at 37 °C, causing decrease in pH and increase in PaO2 and PaCO2. If technicians are aware of the patient’s temperature, they will correct the pH for the patient’s temperature and report a more alkalotic pH. Do not titrate respiration or management based on corrected pH because it should be alkalotic in significantly hypothermic patients to maintain the transmembrane pH potential. 22
  23

Management of dysrhythmias in hypothermia

- **Osborn wave**
  ○ **commonly associated with hypothermia**
  ○ a positive deflection at the J point in precordial and limb leads
  ○ size correlates with the degree of hypothermia
- All intervals will be prolonged due to slowed impulse conduction through K+ channels 24
- Sinus bradycardia: Rewarm
- Atrial fibrillation with slow ventricular response: Rewarm
- Ventricular Tachycardia and ventricular fibrillation: Defibrillation at 200J once. If the rhythm continues to be ventricular tachycardia or ventricular fibrillation defer further shocks, adrenaline, or medications until T > 30°C (86°F) 25,26
- Asystole: Start ACLS, rewarm, initiate ECMO

Thoracic Cavity Irrigation

- Place anterior/superior (anterior midclavicular line at 2^nd^ to 4th intercostal space) and posterior/inferior (posterior axillary line at 5^th^ or 6^th^ /intercostal space) chest tubes on either side of the chest^27^
- Use a high-rate infuser to deliver heated IV bags through the anterior chest tube
- Monitor for signs of tension hydrothorax and allow adequate time for fluid to drain through the posterior chest tube

Termination of resuscitation efforts

Termination of resuscitation can be considered when

- K > 12 mmol 28
- Resuscitation shows no improvement, and the patient is >32°C

**Closed Loop Communication**

▪ Pointed roles during a critical event are extremely important in providing maximal care
▪ Address team members by their roles or names when asking for an action and request a verbal confirmation that you were heard and understood

## SIMULATION ASSESSMENT

*Drowning Complicated by Hypothermia*

Learner: _________________________________________

***Assessment Timeline***

This timeline is to help observers assess their learners. It allows observer to make notes on when learners performed various tasks, which can help guide debriefing discussion.[Table t15-jetem-10-1-s43]

**Critical Actions:**

□ Place patient on monitor and obtain a full set of vitals, including temperature
□ Obtain point-of-care glucose
□ Place two large bore IV catheters
□ Initiate heated high-flow oxygen delivery
□ Remove all wet clothing
□ Place a Bair-hugger
□ Perform a physical exam specifically evaluating for potential traumatic injuries
□ Place a temperature-sensing foley catheter
□ Place two right-sided chest tubes for thoracic irrigation
□ Update partner on diagnosis and planned disposition
□ Transfer to a medical ICU with ECMO capabilities

**Summative and formative comments:**

**Milestones assessment:** [Table t16-jetem-10-1-s43]
