# Supplementary material for: Drowning Complicated by Hypothermia
Source: J Educ Teach Emerg Med. 2025 Jan 31;10(1):S43–74. doi: 10.21980/J8QS7P (PMC11801487; doi:10.21980/J8QS7P)

## Slide 1
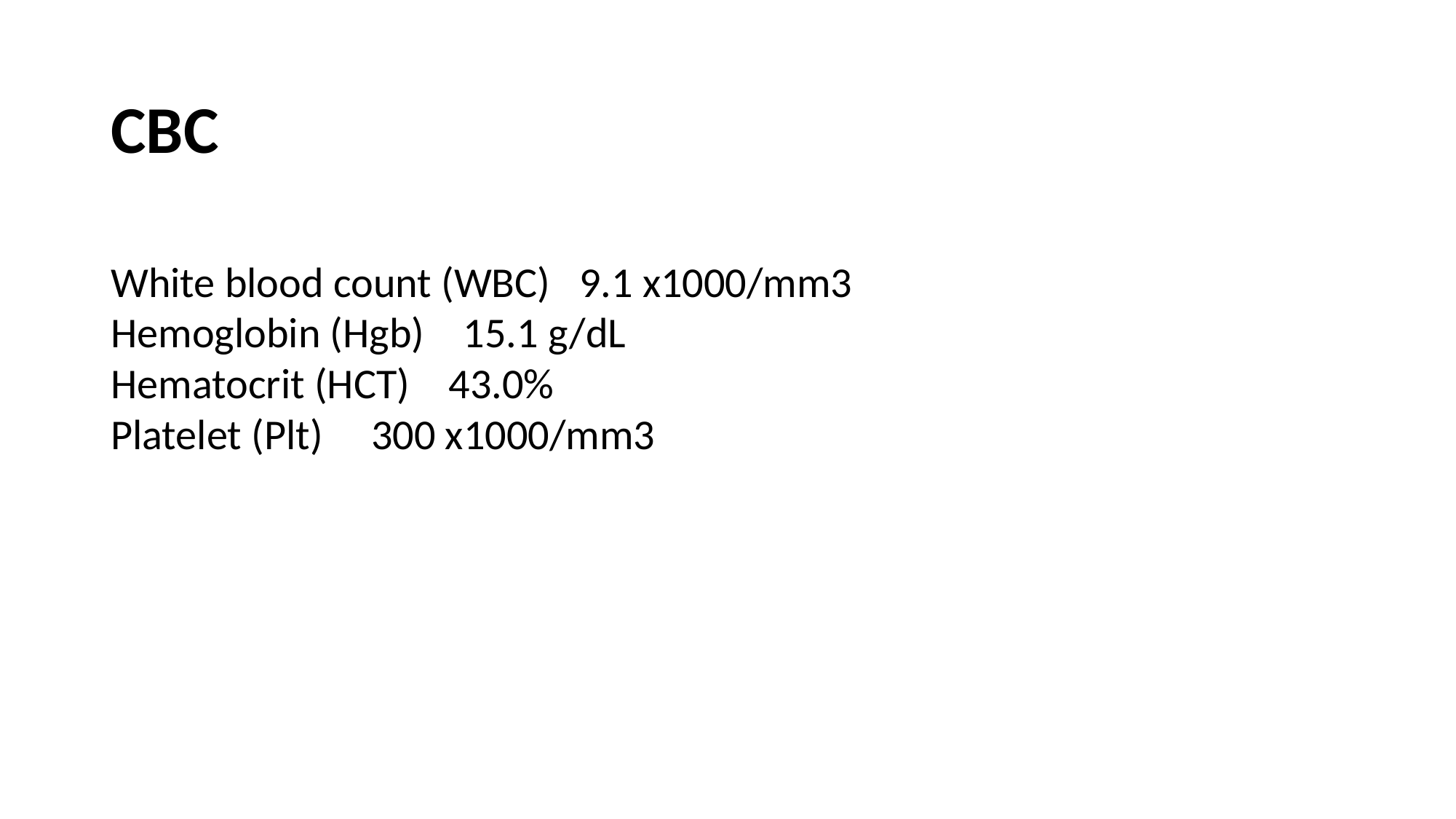

CBC
White blood count (WBC) 9.1 x1000/mm3
Hemoglobin (Hgb) 15.1 g/dL
Hematocrit (HCT) 43.0%
Platelet (Plt) 300 x1000/mm3

## Slide 2
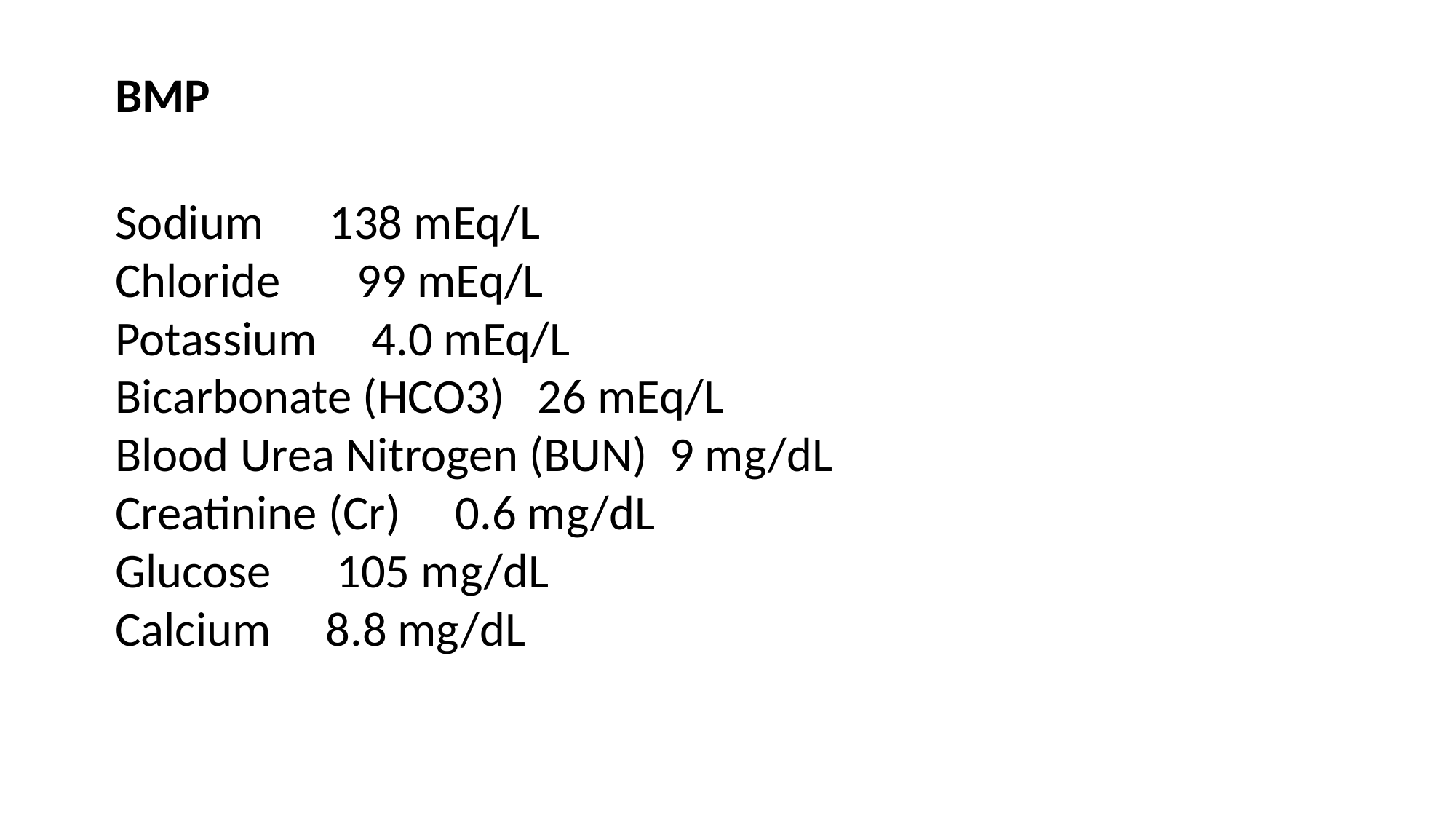

BMP
Sodium 138 mEq/L
Chloride 99 mEq/L
Potassium 4.0 mEq/L
Bicarbonate (HCO3) 26 mEq/L
Blood Urea Nitrogen (BUN) 9 mg/dL
Creatinine (Cr) 0.6 mg/dL
Glucose 105 mg/dL
Calcium 8.8 mg/dL

## Slide 3
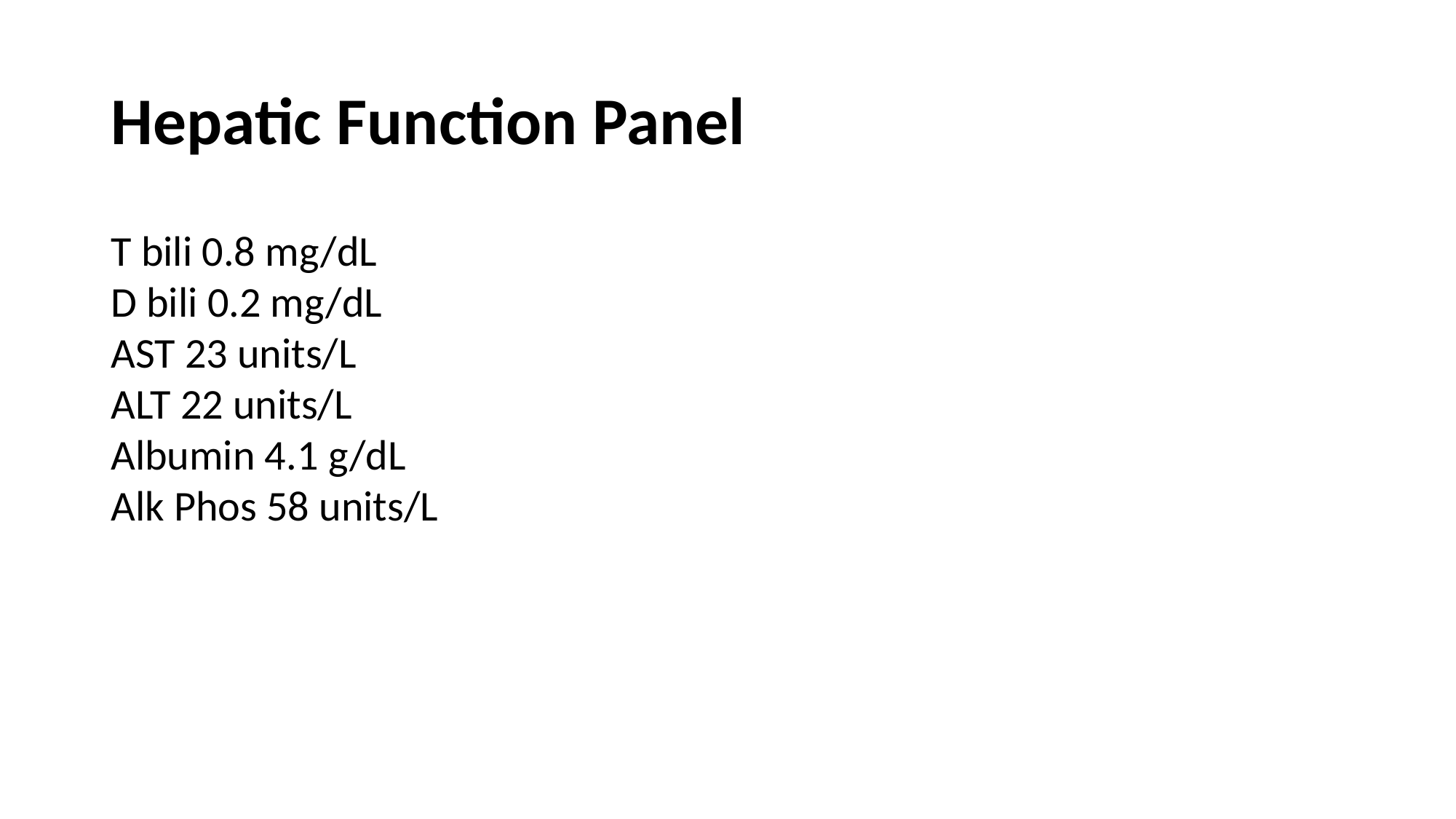

# Hepatic Function Panel
T bili 0.8 mg/dL
D bili 0.2 mg/dL
AST 23 units/L
ALT 22 units/L
Albumin 4.1 g/dL
Alk Phos 58 units/L

## Slide 4
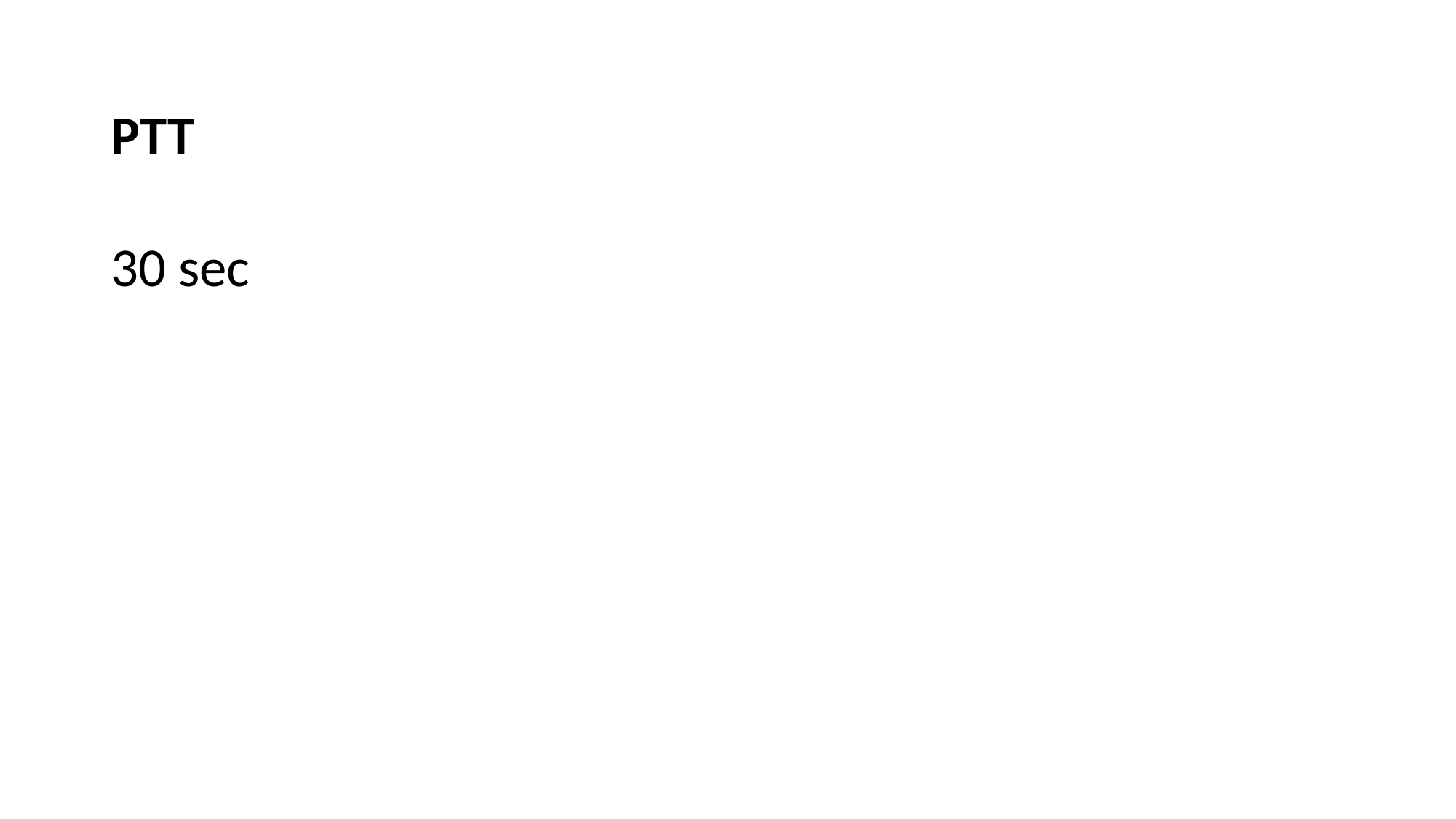

PTT
30 sec

## Slide 5
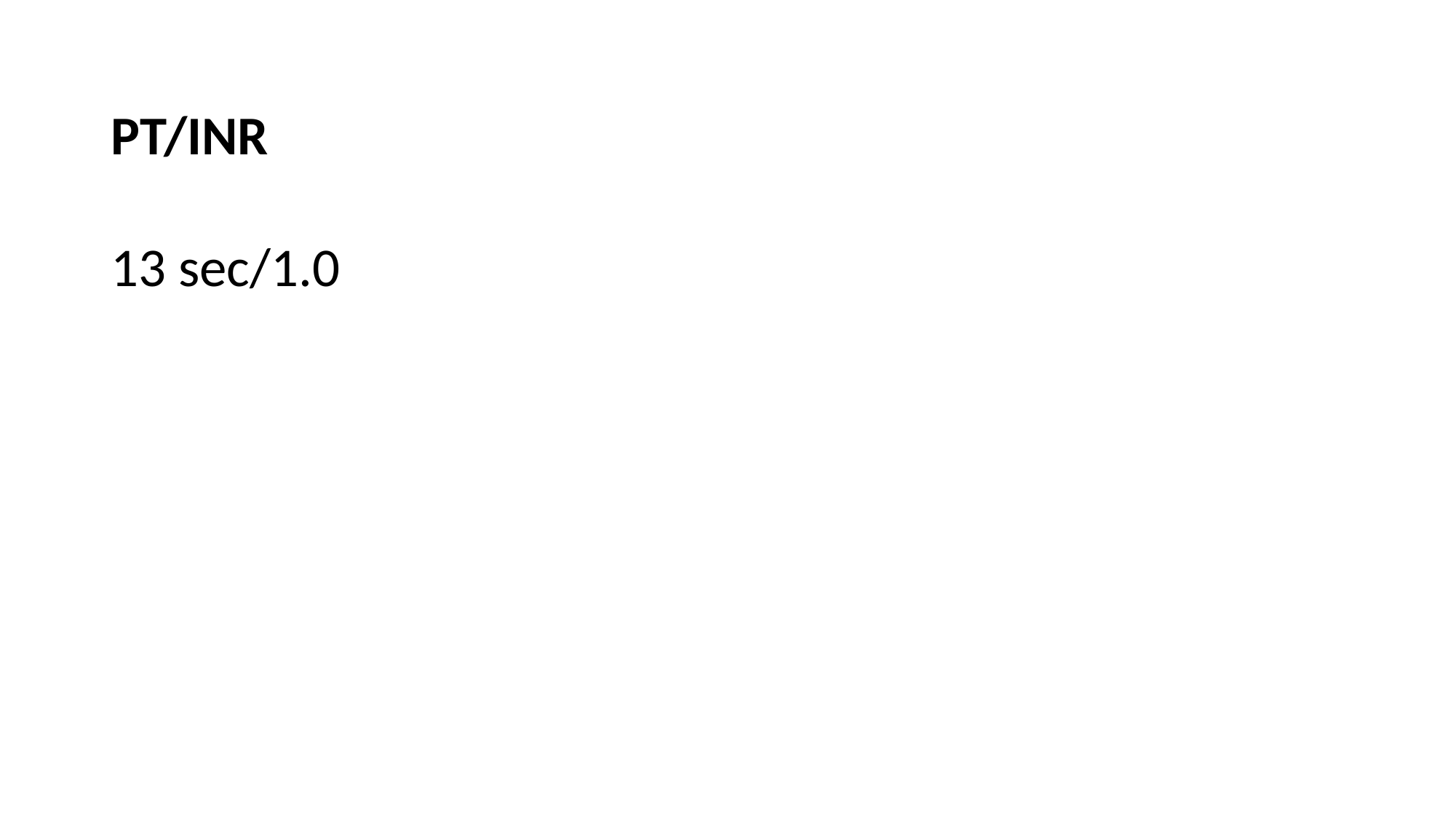

PT/INR
13 sec/1.0

## Slide 6
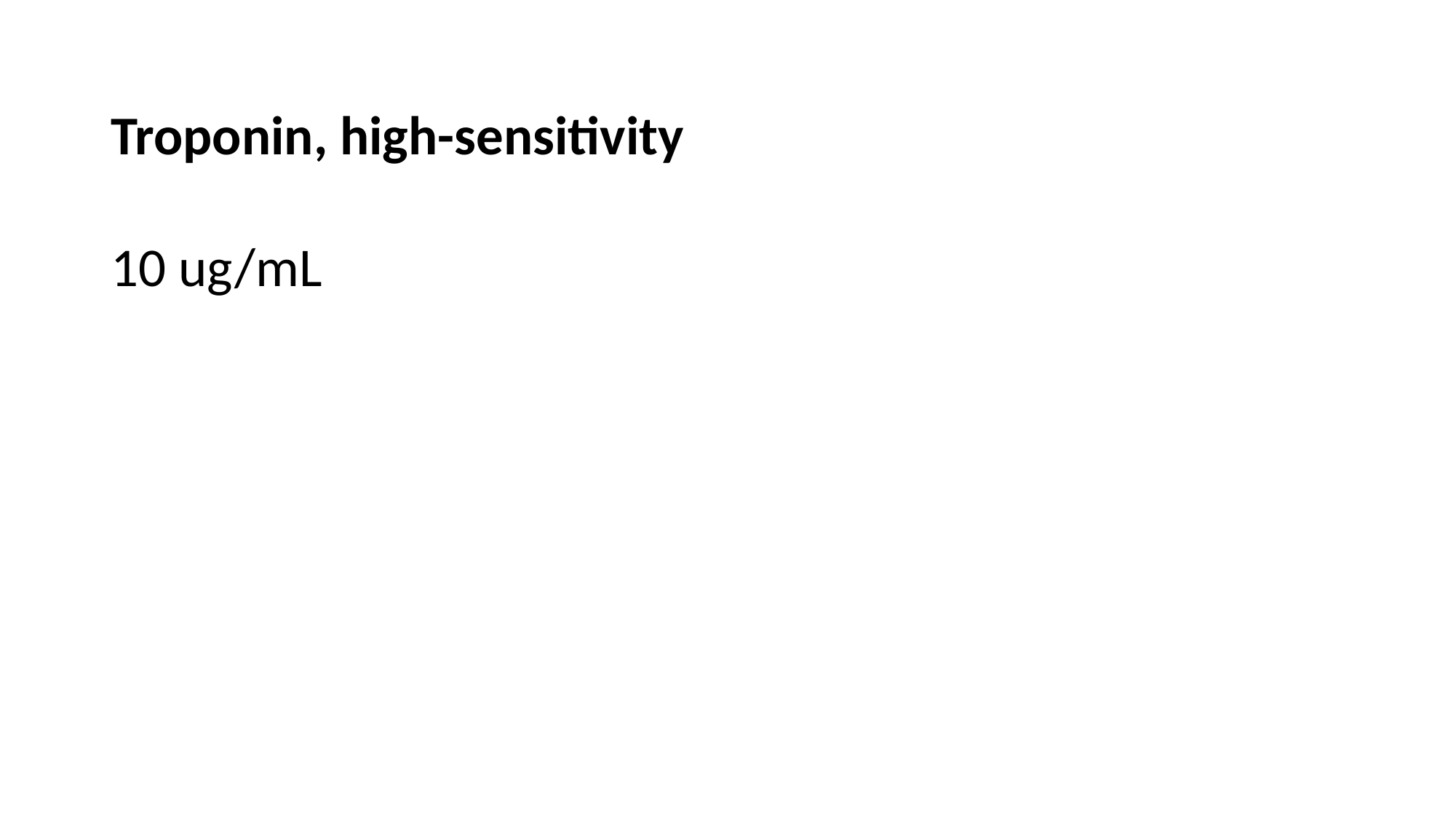

Troponin, high-sensitivity
10 ug/mL

## Slide 7
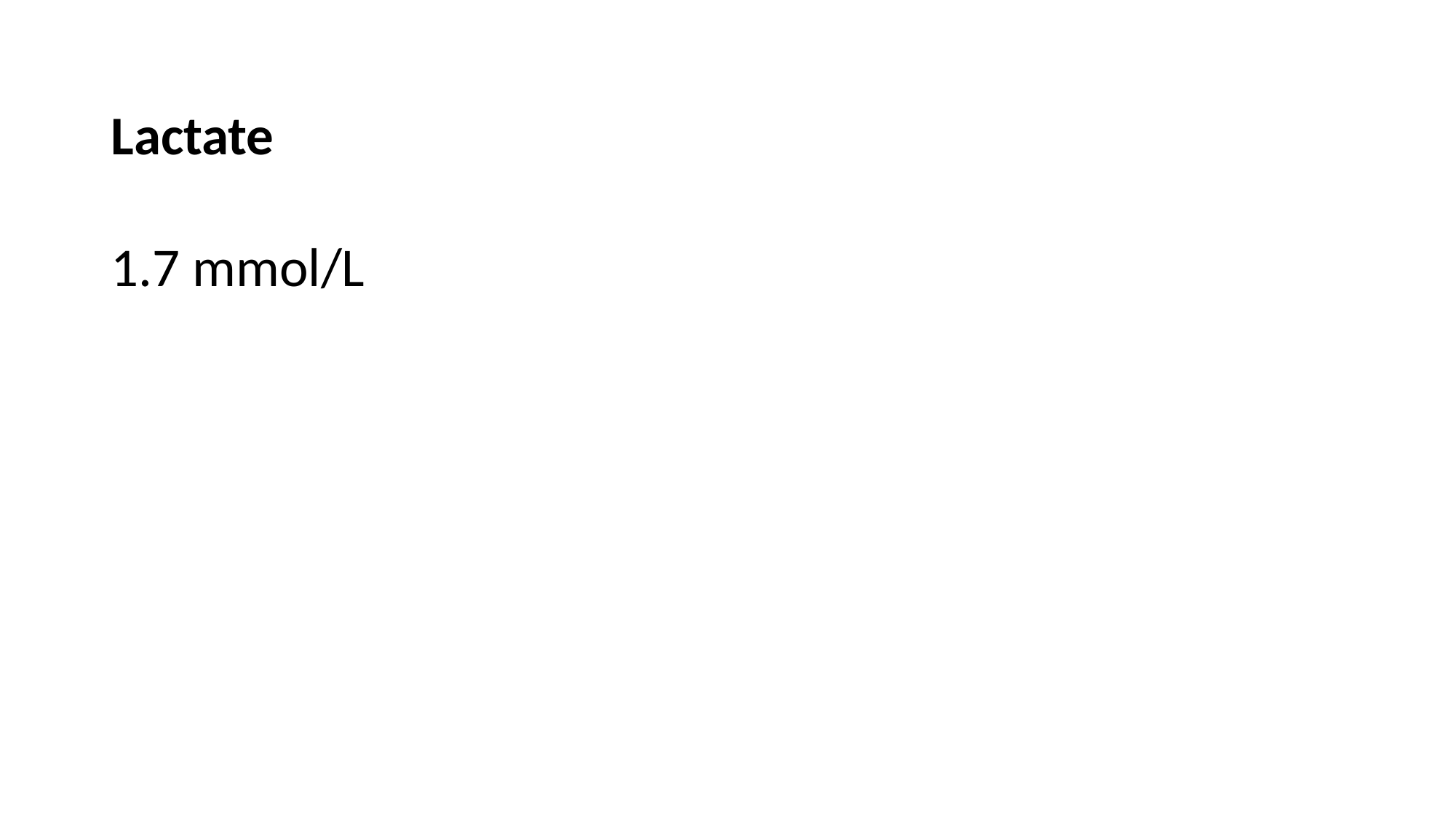

Lactate
1.7 mmol/L

## Slide 8
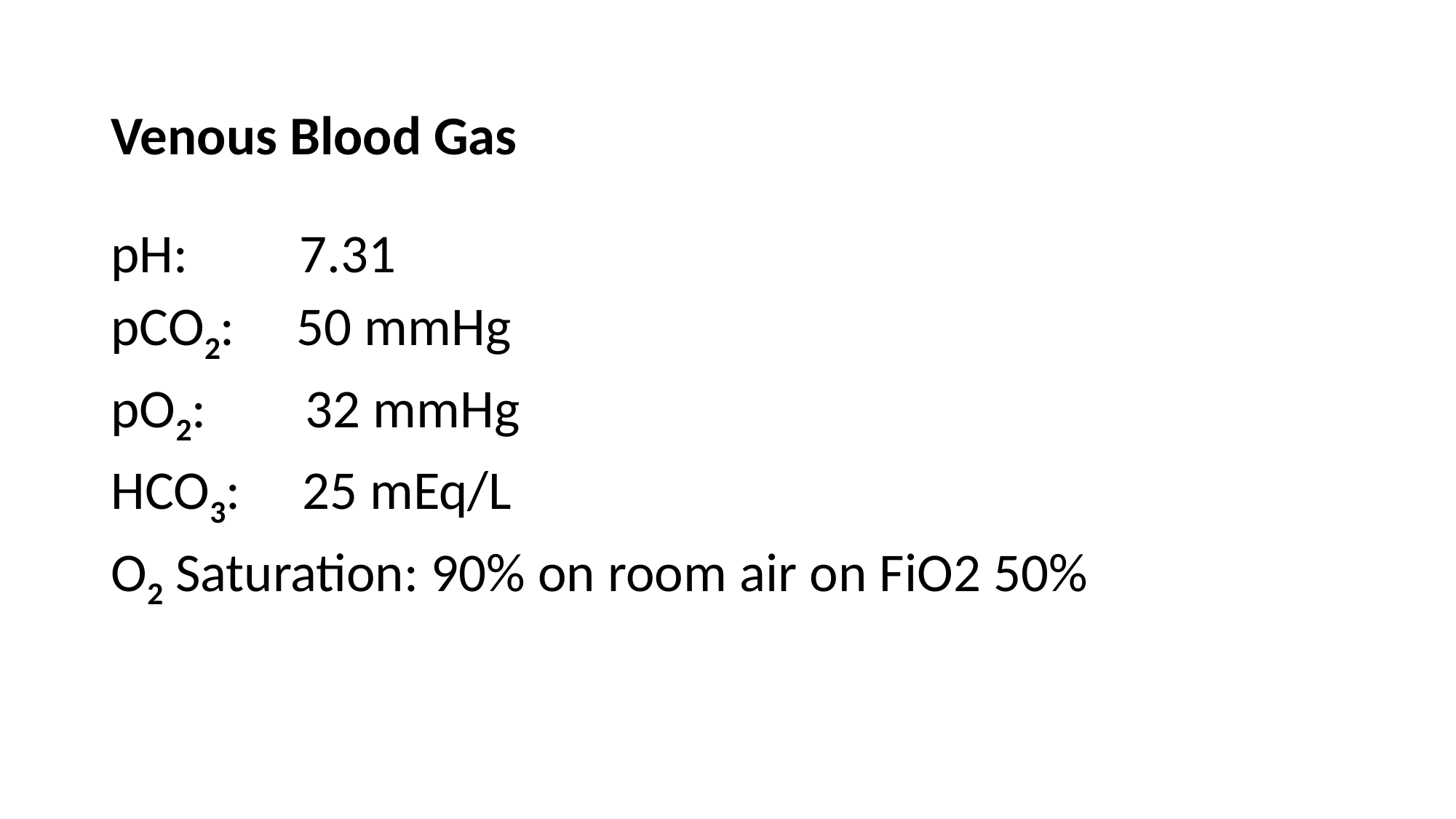

Venous Blood Gas
pH: 7.31
pCO2: 50 mmHg
pO2: 32 mmHg
HCO3: 25 mEq/L
O2 Saturation: 90% on room air on FiO2 50%

## Slide 9
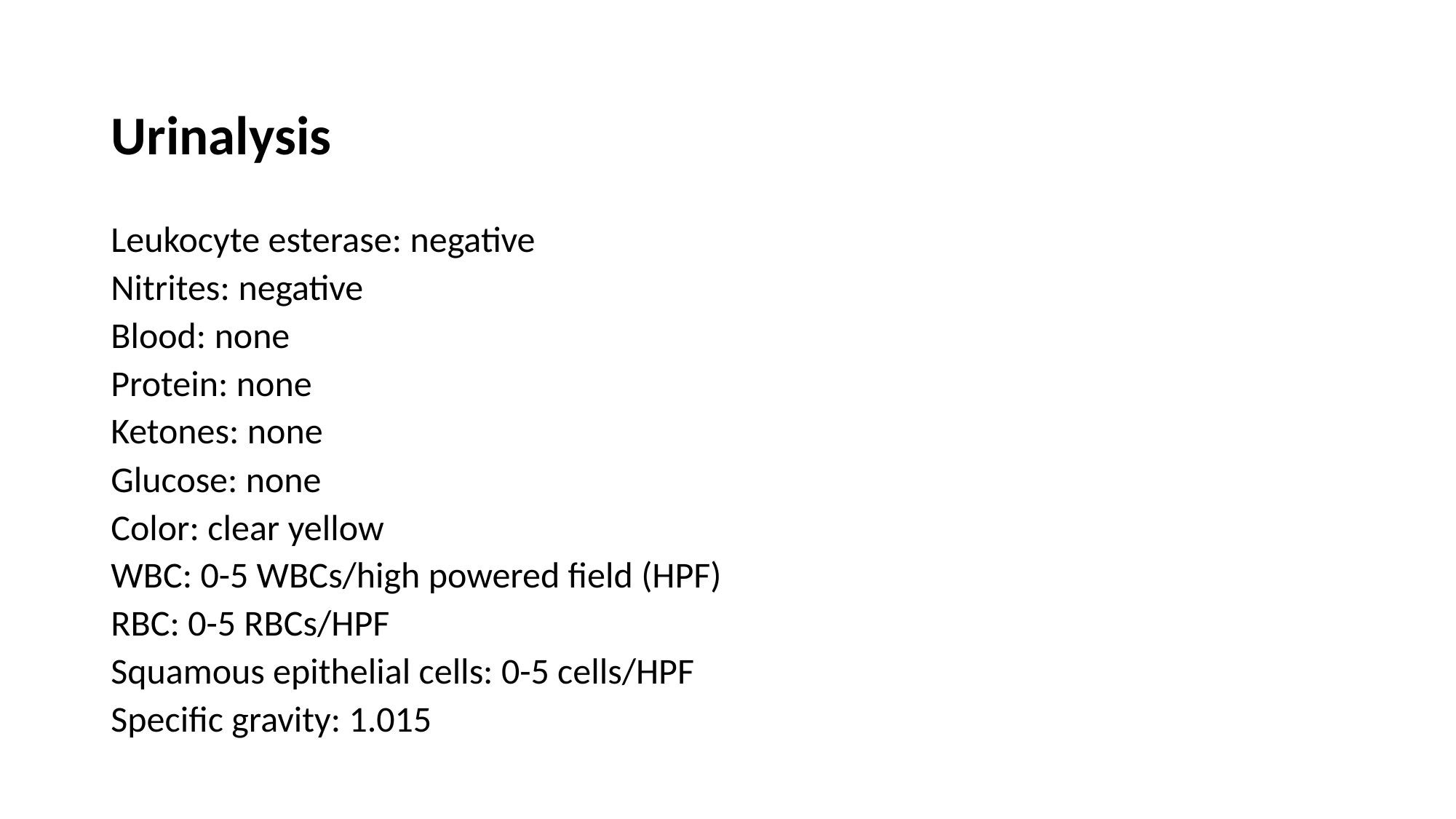

Urinalysis
Leukocyte esterase: negative
Nitrites: negative
Blood: none
Protein: none
Ketones: none
Glucose: none
Color: clear yellow
WBC: 0-5 WBCs/high powered field (HPF)
RBC: 0-5 RBCs/HPF
Squamous epithelial cells: 0-5 cells/HPF
Specific gravity: 1.015

## Slide 10
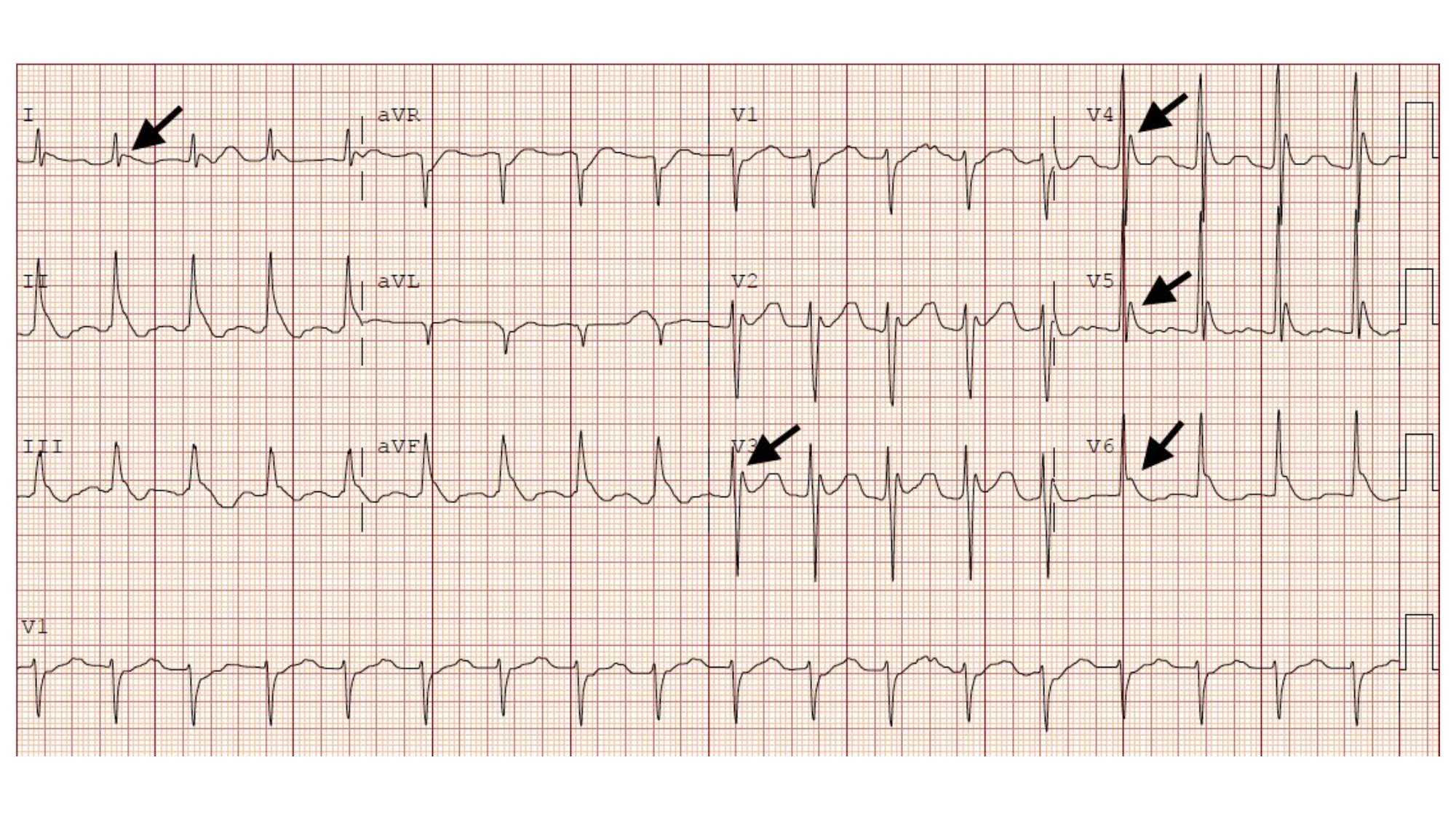

## Slide 11
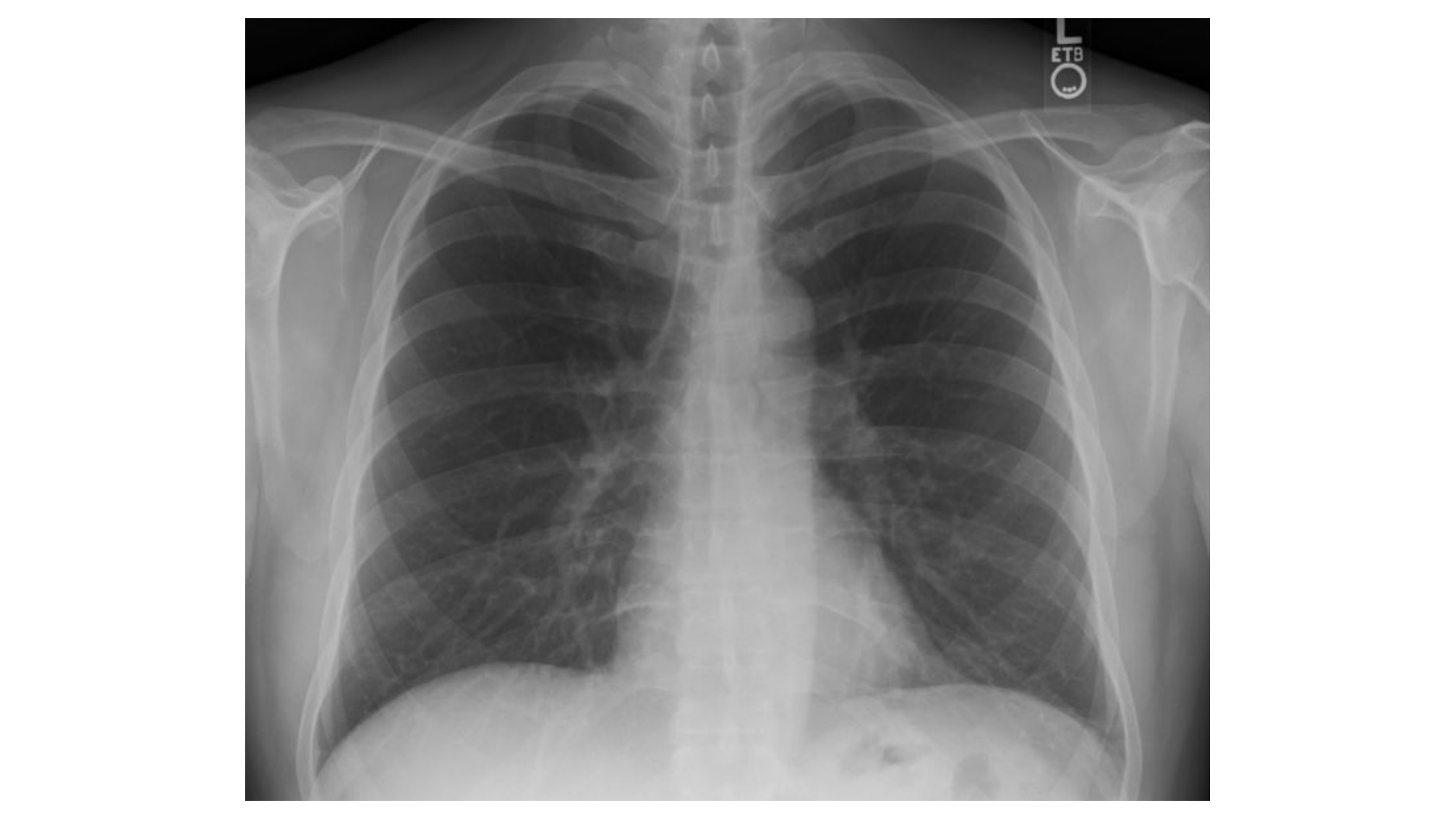

## Slide 12
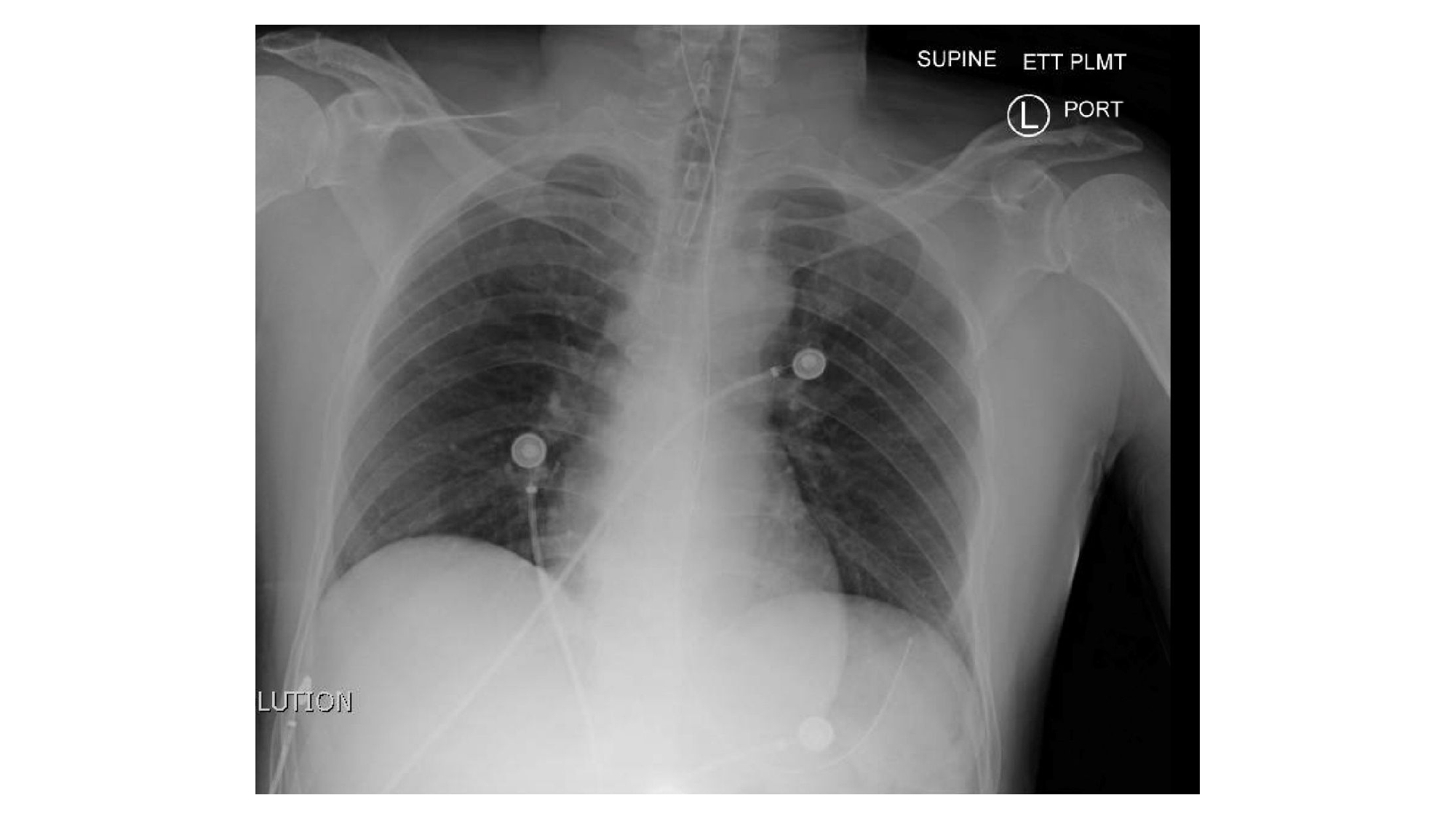

## Slide 13
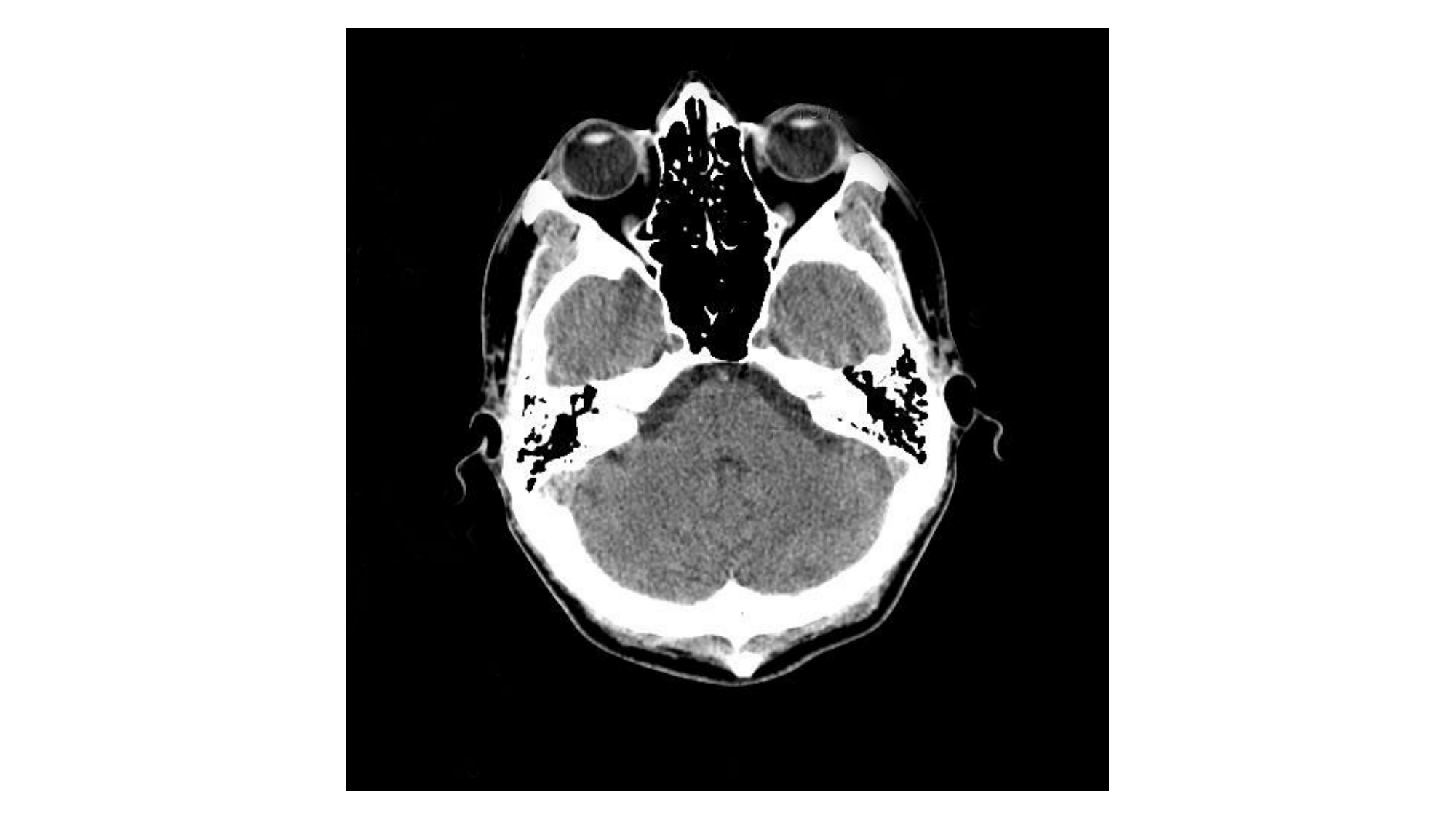

## Slide 14
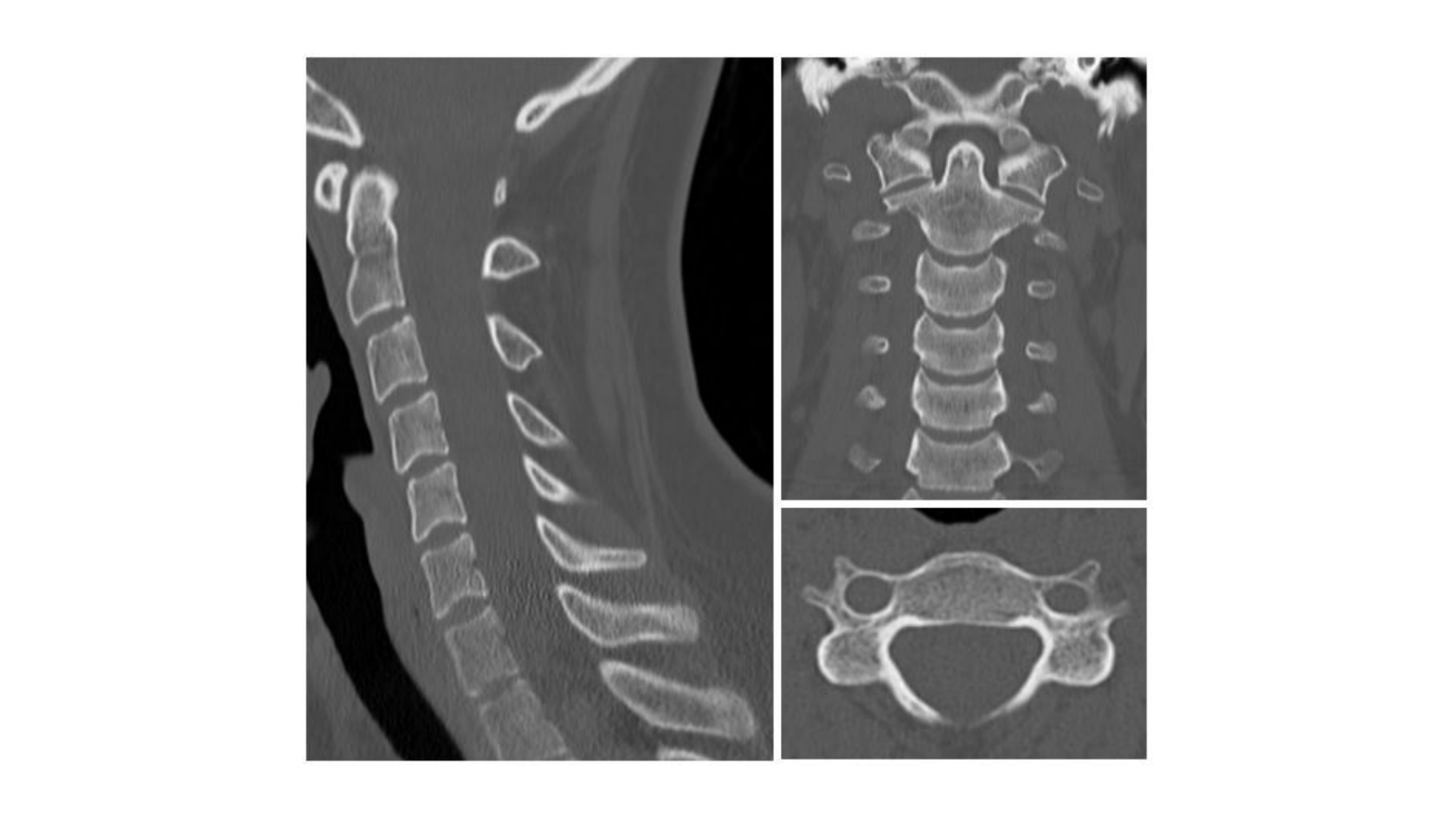

Supplement: Supplementary file 1 [file 10-1-S43-supp1.pptx]
